# AhpC of the mycobacterial antioxidant defense system and its interaction with its reducing partner Thioredoxin-C

**DOI:** 10.1038/s41598-017-05354-5

**Published:** 2017-07-11

**Authors:** Chui Fann Wong, Joon Shin, Malathy Sony Subramanian Manimekalai, Wuan Geok Saw, Zhan Yin, Shashi Bhushan, Arvind Kumar, Priya Ragunathan, Gerhard Grüber

**Affiliations:** 1 Nanyang Technological University, School of Biological Sciences, 60 Nanyang Drive, Singapore, 637551 Republic of Singapore; 2 NTU Institute of Structural Biology, Nanyang Technological University, 59 Nanyang Drive, Singapore, 636921 Republic of Singapore

## Abstract

Despite the highly oxidative environment of the phagosomal lumen, the need for maintaining redox homeostasis is a critical aspect of mycobacterial biology. The pathogens are equipped with the sophisticated thioredoxin- (Trx) and peroxiredoxin system, including TrxC and the alkyl hydroperoxide reductase subunit C (AhpC), whereby TrxC is one of the reducing partners of AhpC. Here we visualize the redox modulated dodecamer ring formation of AhpC from *Mycobacterium bovis* (BCG strain; *Mb*AhpC) using electron microscopy and present novel insights into the unique N-terminal epitope (40 residues) of mycobacterial AhpC. Truncations and amino acid substitutions of residues in the unique N-terminus of *Mb*AhpC provide insights into their structural and enzymatic roles, and into the evolutionary divergence of mycobacterial AhpC versus that of other bacteria. These structural details shed light on the epitopes and residues of TrxC which contributes to its interaction with AhpC. Since human cells lack AhpC, the unique N-terminal epitope of mycobacterial AhpC as well as the *Mb*AhpC-TrxC interface represent an ideal drug target.

## Introduction

**Figure 1:**
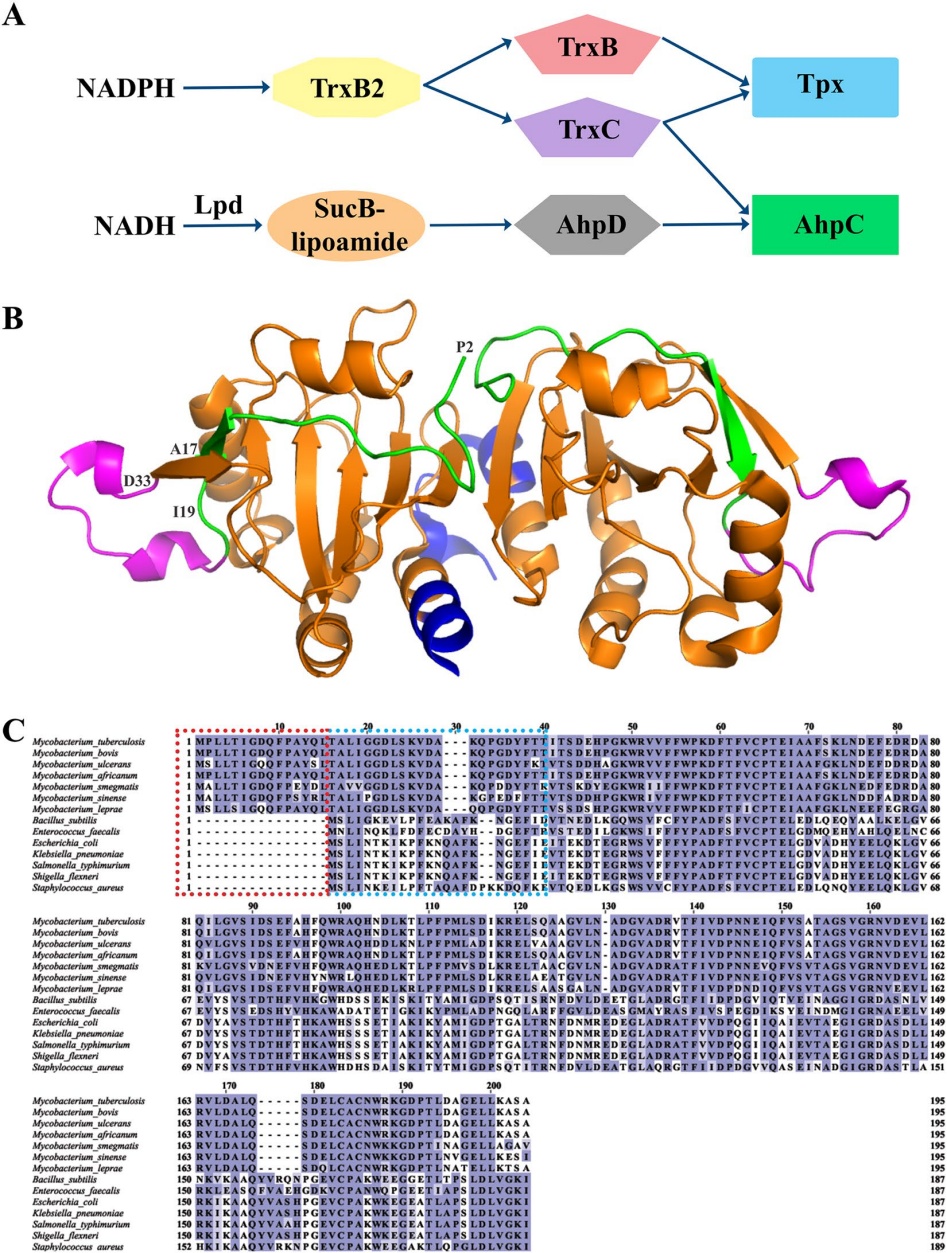
Peroxidatic catalytic pathway of *Mycobacterium*. (**A**) AhpC has various disulfide oxidoreductases such as TrxC and the proposed AhpD. In the former, AhpC has been proposed to function with TrxC in the reduction of ROS. In the latter, an orchestra of AhpD, SucB-lipoamide and LpD is necessary to reduce ROS. In both mechanisms, the reduction by either TrxC or AhpD results in the formation of a disulfide bridge along two cysteine residues of AhpC. (**B**) *Mt*AhpC_C176S_ mutant dimer structure (PDB ID: 2BMX)^13^. The N-terminal residues 1–15 are highlighted in *green*. The extra loop (amino acids 23–34) is shown in *magenta*, and the very C-terminal residues are colored in *blue*. (**C**) Multiple sequence alignment of AhpC from *M*. *tuberculosis*, *M*. *bovis*, *M*. *ulcerans*, *M*. *africanum*, *M*. *smegmatis*, *M*. *sinense*, *M*. *leprae*, *B*. *subtilis*, *E*. *faecalis*, *E*. *coli*, *K*. *pneumonia*, *S*. *typhimurium*, *S. flexneri* and *S*. *aureus* using Clustal-Omega^30^. The stretch of additional residues found in mycobacterial AhpC are highlighted with a *red dashed box*. The residues deleted for the generation of *Mb*AhpC_41–195_ are highlighted with a *blue dashed box*.

Tuberculosis (TB) with its causative agent, *Mycobacterium tuberculosis* (*Mtb*) has claimed many lives^1^ ever since it has been made known to mankind from ancient times. Ranked above HIV/AIDS in causing mortality due to an infectious disease^2^, World Health Organization’s Global Tuberculosis Report 2016 revealed an additional 10.4 million new cases worldwide in 2015^2^. It was also understood that the current TB research and development is still severely unfunded^2^ and that the emergence of resistant strains against the first line drug, isoniazid (INH), is a huge cause of concern^3^. Despite being exposed to numerous oxidative and nitrosative stresses when mounting a pathogenic cycle of infection and transmission, *Mtb* is still able to persist and proliferate through various antioxidant defense systems^4, 5^. These antioxidant defense systems in *Mtb* have led to increased drug resistance and virulence. The susceptibility of *Mtb* to INH results from the activation of the gene *KatG*, encoding the mycobacterial specific catalase-peroxidase that plays a crucial role in evading or countering the host’s respiratory burst by decomposing hydrogen peroxide (H_2_O_2_) into water and oxygen^6^. The deletion of *KatG* in *Mtb* has shown to increase the bacteria’s sensitivity to H_2_O_2_^7^. To ensure its virulence, *Mycobacterium* have evolved an alternative peroxidase system including the alkyl hydroperoxide reductase subunit C (AhpC)^8^ (Fig. 1A). Importantly, when exposed to INH, the INH-resistant *Mtb* exudes a *KatG*-deficiency, but also enhanced expression of *Mt*AhpC characteristic^9^.

Like most bacteria, the mycobacterial AhpC belongs to the typical 2-Cys peroxiredoxins (Prxs)^5^. Prxs are a crucial class of antioxidant enzymes needed to protect the cell from oxidative damages induced by reactive oxygen species (ROS). AhpCs of gram-negative bacteria described so far exist as a basic dimeric unit when it is oxidized and form a decamer-ring under reduced conditions^10, 11^. A peroxidatic cysteine (C_P_) residue in one unit of the dimeric AhpC reacts with H_2_O_2_ to form sulfenic acid. A reducing cysteine (C_R_) residue in the other unit of the dimer then attacks the sulfenic acid to release water^5, 12^. As a result, a disulfide bond is formed between the peroxidatic and reducing cysteine residues. Finally, the oxidized AhpC becomes regenerated by the NADH-dependent oxidoreductase, AhpF^12^. In the case of mycobacterial AhpC, a crystallographic structure of the *Mtb* AhpC mutant C176S (*Mt*AhpC_C176S_)^13^ revealed that the asymmetric unit contains a dimer and a monomer. It was proposed that *Mt*AhpC_C176S_ would form a dodecamer-ring in solution^13^ instead of a decamer as described for the AhpC of gram-negative bacteria^10, 11, 14^. Connected with this diversity, the mycobacterial antioxidant system is not equipped with a gene encoding the oxidoreductase AhpF. Instead of AhpF, AhpC is reduced by the mycobacterial specific alkyl hydroperoxidase subunit D (AhpD)^15^ (Fig. 1A) or the thioredoxin TrxB and TrxC (TrxB, TrxC)^16^, as well as thioredoxin reductase TrxB2, although the role of the reduction through the thioredoxin system is still a debate^1, 15^ and its interaction epitopes are not resolved yet. It may reflect the difference of mycobacterial AhpCs to related typical 2-Cys peroxiredoxins by employing three rather than two cysteine residues involved in catalysis^13^.

Interestingly, the dimer structure of *Mt*AhpC_C176S_ mutant^13^ (Fig. 1B) as well as a protein sequence analysis of mycobacterial AhpCs revealed differences in the amino acid composition of AhpCs from other sources (Fig. 1C), in particular the presence of a unique stretch of 15 amino acid residues and the secondary structure of its N-terminal 40 residues. This N-terminal stretch, highlighted in green in Fig. 1B, is proposed to span one AhpC molecule and thereby bridging the two oligomeric interfaces, in which the catalytic C_P_ and C_R_ of the AhpC-ring are located. In addition, a crystal structure alignment with *Ec*AhpC revealed that *Mt*AhpC_C176S_ forms an additional loop in the N-terminus from residues 23–34 (Fig. 1B, *magenta*). Elucidating the molecular mechanism and architecture of mycobacterial AhpC as well as its interaction with AhpD and TrxC in solution will shed light on the potential differences in function and regulation of the unique mycobacterial thioredoxin- and peroxiredoxin system.

Here we used 2-dimensional (2D) projection analysis of electron micrographs to visualize the dodecamer ring of reduced wild-type (wt) AhpC from *M*. *bovis* (BCG strain; *Mb*AhpC), whose amino acid sequence is identical to *Mtb*, and to demonstrate the oligomeric formation of *Mb*AhpC due to redox-modulation. Using NMR-titration experiments of labeled *Mb*TrxC with *Mb*AhpC, the epitope to which *Mb*AhpC binds in *Mb*TrxC is presented and opens the door for an ideal drug target. Truncations and amino acid substitutions of residues in of the unique N-terminus of *Mb*AhpC provide insights into their structural and enzymatic roles.

## Results

### Production, purification and characterization of recombinant MbAhpC

**Figure 2:**
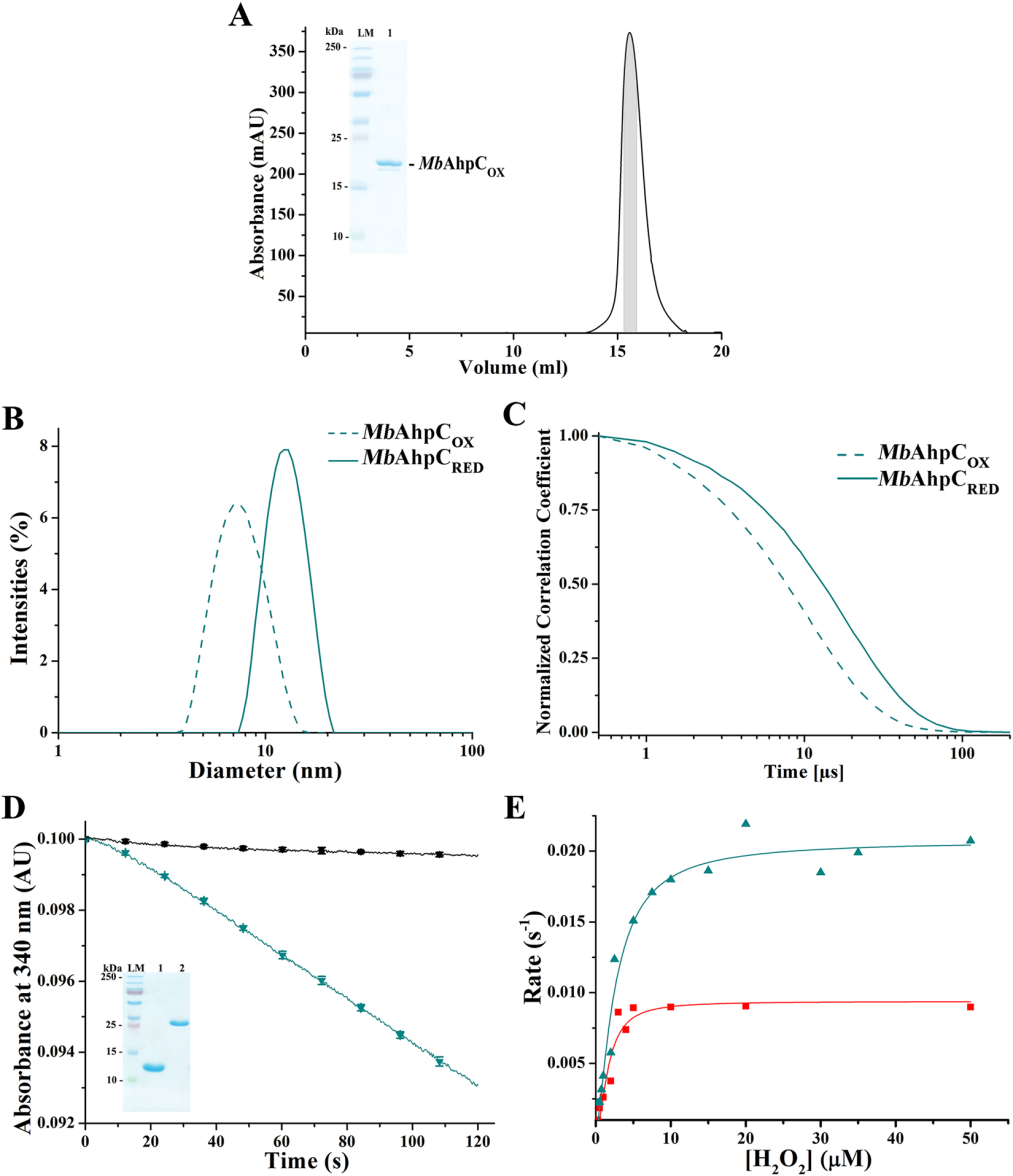
Protein characterization of *Mb*AhpC. (**A**) The recombinant and oxidized *Mb*AhpC eluted at approximately 16 ml on a Superdex 200 HR 10/30 column and showed high purity on a 17% SDS gel (*inset*). (**B**) DLS analysis of the oxidized (*green dashed line*) and reduced (*green solid line*) *Mb*AhpC. (**C**) Normalized correlation function of DLS studies of *Mb*AhpC in 50 mM Tris/HCl, pH 7.5, 200 mM NaCl. A slowed relaxation of the autocorrelation curve in reduced *Mb*AhpC (*green solid line*) was observed as compared with oxidized *Mb*AhpC (*green dashed line*). (**D**) NADPH-oxidation of *Mb*TrxC and *Mb*AhpC. The control experiment was performed in the absence of enzymes and H_2_O_2_ (*black solid line*). The utilized *Mb*TrxC (*lane 1*) and *Mb*TrxB2 (*lane 2*) showed high purity on a 17% SDS gel (*inset*) after size exclusion chromatography purification. The size of *Mb*TrxC is 12,203 Da and *Mb*TrxB2 is 35,643 Da. A significant drop in absorbance was observed due to the presence of wt *Mb*AhpC (*green solid line*). (**E**) The Michaelis-Menten plot of *Mb*AhpC and *Mb*AhpC_T5A/D8A_ were performed by fitting data of at least ten concentrations of H_2_O_2_ to establish the enzymatic kinetic parameters of wt *Mb*AhpC and *Mb*AhpC_T5A/D8A_. *Mb*AhpC showed a higher enzymatic efficiency (*green line and triangle symbols*) as compared to *Mb*AhpC_T5A/D8A_ (*red line and square symbols*) whereby the reaction approached V_max_ rapidly in the mutant as compared to wt *Mb*AhpC.

Recombinant *Mb*AhpC was purified in oxidized conditions by affinity- and size exclusion chromatography as described in Materials and Methods. The pure protein eluted at 16 ml on a Superdex 200 HR 10/30 column in the absence of the reducing agent DTT (oxidized condition), corresponding to a dimeric species (Fig. 2A). To confirm the observation of size exclusion chromatography (SEC), DLS experiments with *Mb*AhpC were performed. As shown in Fig. 2B and C, the DLS profile revealed a dimeric species with an estimated molecular weight of 59.9 ± 22.6 kDa (26.2% polydispersity), and thus, confirming the SEC result described above.

To examine the enzymatic activity of *Mb*AhpC, the *Mb*TrxB2-TrxC system composed of TrxB2 and TrxC (Fig. 1A) was utilized, and both proteins were generated and purified for the peroxidase assay (see Material and Methods and *inset* of Fig. 2D). In this assay, NADPH-oxidation by TrxC provides the electrons via TrxB2 to *Mb*AhpC for H_2_O_2_ reduction. As shown in Fig. 2D, *Mb*AhpC reacted with 50 μM H_2_O_2_ and actively oxidized NADPH, as compared to the profile of the control measurement in which no *Mb*AhpC was injected. To further characterise the enzymatic traits of *Mb*AhpC, the Michaelis-constant (*K*_*m*_) of 2.57 ± 0.36 µM and the catalytic turnover number (*k*_*cat*_) of 0.021 ± 0.001 s^−1^ were determined (Fig. 2E, Table 1), resulting in a catalytic efficiency of *Mb*AhpC (*k*_*cat*_ */K*_*m*_ (H_2_O_2_)) of 8.17 × 10^3^ M^−1^ s^−1^. In addition, the enzymatic parameters of *Mb*AhpC with *tert*-butyl hydroperoxide (*t*-bOOH) as a substrate and NADPH as electron donor were determined with a *K*_*m*_ of 2.23 ± 0.49 µM, a *k*_*cat*_ value of 0.027 ± 0.003 s^−1^, and a *k*_*cat*_ */K*_*m*_ of 12.20 × 10^3^ M^−1^ s^−1^ (Table 1).

**Table 1:** Kinetic parameters of mycobacterial AhpC with TrxC and NADPH.

Protein	*k*_*cat*_ ± SD (s^−1^)	*K*_*m*_ ± SD (μM)	*k*_*cat*_ */K*_*m*_ (M^−1^s^−1^)
**H**_**2**_ **O**_**2**_ **as substrate**			
*Mb*AhpC + *Mb*TrxC	0.021 ± 0.001	2.57 ± 0.36	8.17 × 10^3^
*Mb*AhpC_T5A/D8A_ + *Mb*TrxC	0.009 ± 0.001	1.65 ± 0.32	5.44 × 10^3^
***t***-**BOOH as substrate**			
*Mb*AhpC + *Mb*TrxC	0.0272 ± 0.003	2.23 ± 0.49	12.20 × 10^3^

### Ring formation of *Mb*AhpC and its enzymatic traits under reduced conditions

**Figure 3:**
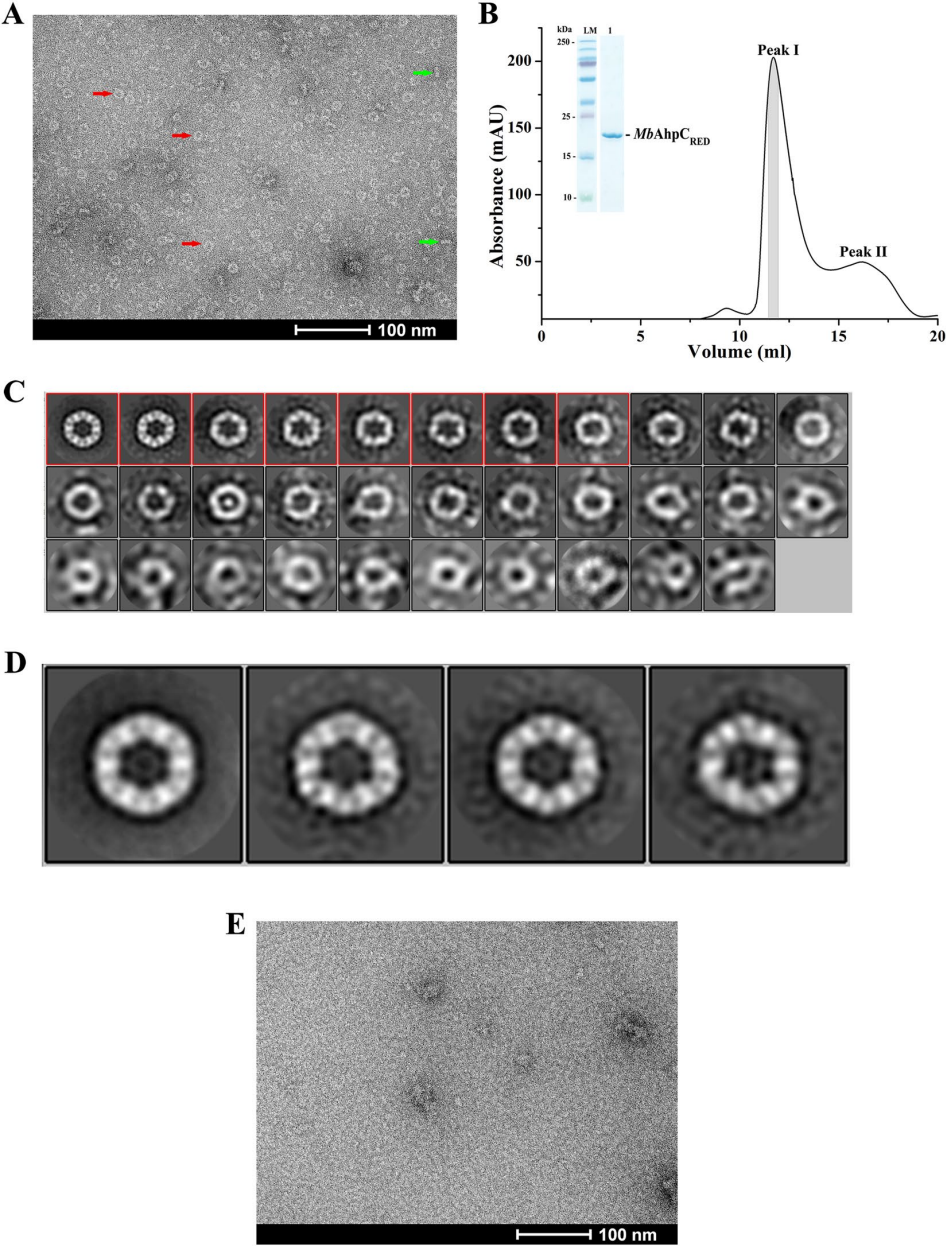
Negative stain electron micrographs of *Mb*AhpC. (**A**) Micrograph of *Mb*AhpC in its reduced form showed ring and rod shape particles, which were likely representing the top (*red arrow*) and side (*green arrow*) views of the ring structures, respectively. (**B**) The recombinant and reduced *Mb*AhpC eluted with majority of the protein fractions at approximately 12 ml on a Superdex 200 HR 10/30 column and showed high purity on a 17% SDS gel (*inset*). (**C**) 2D classification of *Mb*AhpC particles in its reduced form. Raw particles were classified in 32 classes employing 2D classification procedure of RELION image processing package. (**D**) Projection analysis revealed a dodecameric *Mb*AhpC. Particles from the boxed classes in *C* were further classified into 4 classes to improve the quality of 2D classification. (**E**) The ring-shaped particles seen in the micrographs of reduced *Mb*AhpC above were absent in the micrograph of the oxidized protein.

In order to visualize and to define the presence of an oligomeric form of *Mb*AhpC under reduced conditions, electron micrographs of reduced *Mb*AhpC were collected. Images of the reduced enzyme showed considerable amounts of ring-like and rod-shaped particles, likely representing the top and side views of the ring structure, respectively (Fig. 3A). A total of 37 micrographs were subjected to two rounds of 2-dimensional (2D) classifications in RELION (Fig. 3C). Particles were sorted into 32 different classes in the first round to discard bad particles. A total of 3744 particles (eight 2D classes with clear features) selected from the first round of 2D classification were further averaged into four different classes during a second round of 2D classification to improve the image contrast. The averaged projection images show 12 defined masses, indicating that *Mb*AhpC forms a dodecameric-ring under reducing conditions (Fig. 3D). The particles have a diameter of approximately 155 Å, and it is consistent with the reported 140 Å diameter of *Mt*AhpC_C176S_ mutant structure^13^, which reveals that six molecules (A–F) in the asymmetric unit form a half-ring conformation and a dodecameric ring is generated by its crystallographic two-fold symmetry operation (A′–F′).

The SEC-elution profile of reduced (2 mM DTT) *Mb*AhpC revealed a major peak at the column volume of 12 ml (Fig. 3B, peak I) and a minor peak at about 16 ml (Fig. 3B, peak II). As shown by the SDS-PAGE, both peak I (*inset* of Fig. 3B) and peak II contain *Mb*AhpC, indicating that peak I contains an oligomeric form of *Mb*AhpC, and peak II represents a smaller fraction of the dimeric *Mb*AhpC. To confirm this, a DLS experiment was performed using the protein eluted at peak I. The DLS profile of the peak I fraction revealed that reduction of the recombinant protein led to the formation of a higher molecular weight species of about 226.0 ± 55.9 kDa (20.7% polydispersity) (Fig. 2B) with slower diffusion properties, and hence slower relaxation of the autocorrelation function (Fig. 2C), confirming the SEC interpretation above.

In contrast to the reduced *Mb*AhpC, no ring-shaped particles were observed in the electron micrographs of the oxidized *Mb*AhpC protein (Fig. 3E). This confirms the results obtained in the SEC and DLS experiments described above (Fig. 2A–C), that oxidized *Mb*AhpC forms a dimer, and reduction of the enzyme results in a defined dodecameric ring structure of *Mb*AhpC.

### The N-terminal residues are essential for mycobacterial AhpC

**Figure 4:**
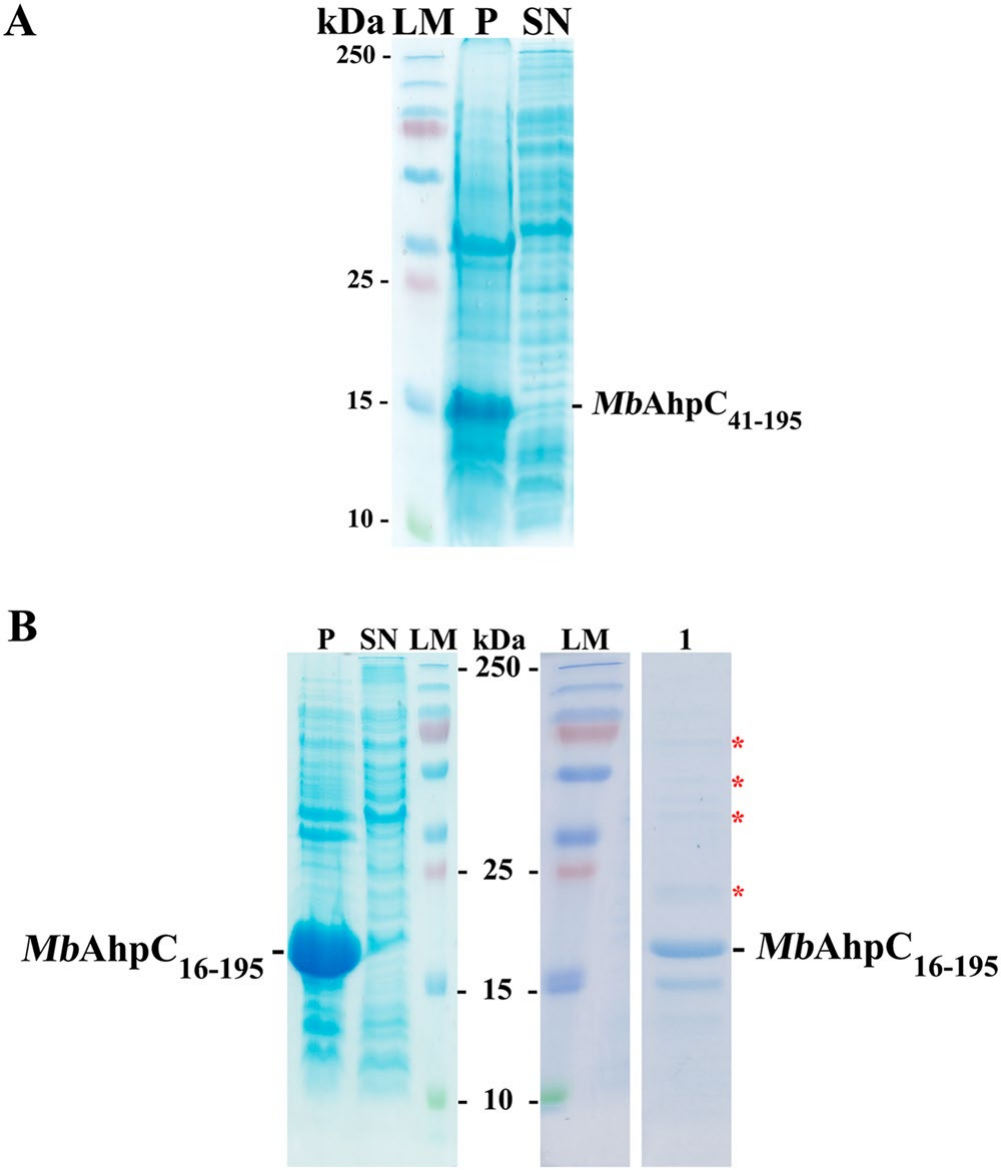
Importance of N-terminal residues. (**A**) Solubility assay of *Mb*AhpC_41–195_ showed low solubility of the protein as reflected in the pellet, labelled as P. SN represents supernatant. (**B**) *Mb*AhpC_16–195_ also showed a less soluble protein (*left*) and the presence of a laddering pattern (*right*) in the 125 mM imidazole fraction after affinity chromatography, indicates the presence of undefined oligomers. LM represents a molecular weight standard. Both SDS gels revealed the importance of the N-terminal residues, particularly from residues 1–15, in maintaining the solubility and oligomerization of *Mb*AhpC.

Figure 1B and C reveal the unique stretch of the residues 1–40 at the very N-terminus of mycobacterial AhpC. To understand whether this unique stretch is critical for the structure and/or the enzymatic traits of the protein, a mutant with deletion of residues 1–40 was genetically engineered (*Mb*AhpC_41–195_). Despite good production of the recombinant *Mb*AhpC_41–195_ mutant in the induction assay, the mutant showed lower protein solubility when compared with wt *Mb*AhpC (Fig. 4A), indicating the importance of the unique N-terminal stretch in protein solubility and stability.

To further determine the critical residues in the N-terminus for the stability of *Mb*AhpC, the N-terminal deletion mutant *Mb*AhpC_16–195_ was genetically engineered. High amount of recombinant *Mb*AhpC_16–195_ was produced however, the amount of soluble protein derived from 6 g of cells was low (Fig. 4B). As shown in the SDS gel after Ni-NTA chromatography (Fig. 4B), ladder formation of protein bands in the SDS gel were observed, reflecting that the protein is not stable and tends to form undefined oligomers (Fig. 4B). These results suggest that the N-terminal deletions may affect the defined oligomeric state of mycobacterial AhpC and its overall stability.

### The importance of the N-terminal residues 23–34 in activity and assembling of *Mb*AhpC

**Figure 5:**
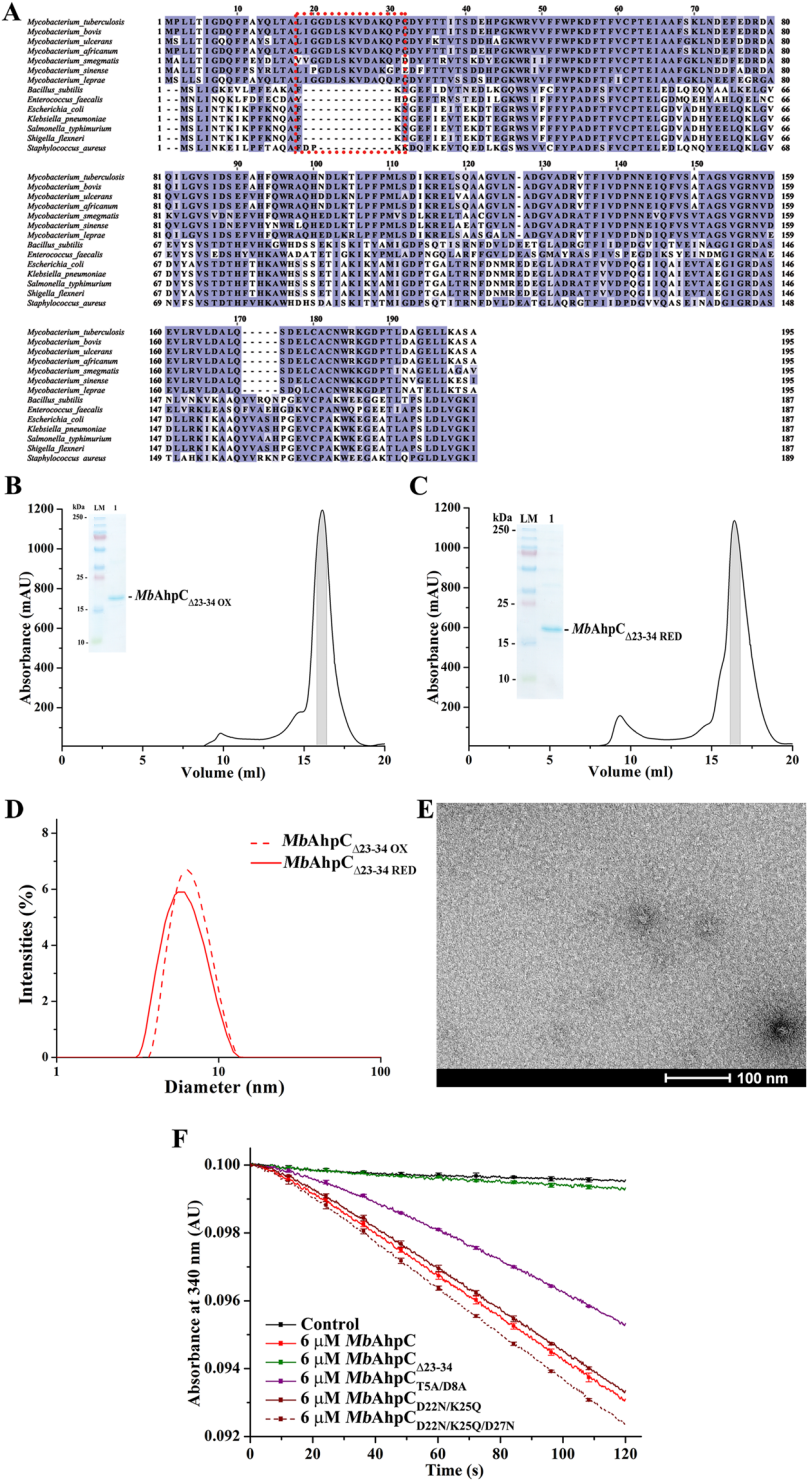
(**A**) Multiple sequence alignments of AhpC from different bacteria using ClustalW^17^. As shown, various *Mycobacterium sp*. presented extra 15 residues located at the N-terminus (*red dashed box*), which were absent in other bacteria species. Mutations with deletion from residues 23–34 were constructed and both oxidized (**B**) and reduced (**C**) mutants eluted at approximately 16 ml on a Superdex 200 HR 10/30 column and showed high purity on a 17% SDS gel (*inset*). (**D**) DLS analysis of oxidized (*red dashed line*) and reduced (*red solid line*) of *Mb*AhpC_Δ23–34_. Both redox states of the protein revealed a similar diameter unlike that of wt *Mb*AhpC. (**E**) Negative stained EM-images of reduced *Mb*AhpC_Δ23–34_. The lack of ring-shaped particles in the micrograph indicates the inability of *Mb*AhpC_Δ23–34_ to form an oligomeric ring. (**F**) NADPH-oxidation of *Mb*AhpC, -AhpC_Δ23–34_, -AhpC_T5A/D8A_, -AhpC_D22N/K25Q_ or -AhpC_D22N/K25Q/D27N_ measured at 50 µM H_2_O_2_ is shown as a representative to highlight the effects of the mutations generated. A control without *Mb*AhpC is represented with a *black solid line*. The activity of wt *Mb*AhpC is indicated with a *red solid line*. The deletion of residues 23–34 (*green solid line*) altered the activity of the protein, hence, showing activities close to that of which resembles the background activity. However, the mutations in T5 and D8 showed the decline of activity of *Mb*AhpC_T5A/D8A_ as shown with the *purple solid line*. On the other hand, the enzymatic activity of *Mb*AhpC_D22N/K25Q_ (*brown solid line*) or *Mb*AhpC_D22N/K25Q/D27N_ (*brown dashed line*) have similar activity to wt *Mb*AhpC.

As shown by the multiple sequence alignments of AhpCs from different bacteria species using the program ClustalW^17^, a stretch of additional residues was observed in the N-terminus of mycobacterial AhpC and predicted to form an extra loop in the N-terminus of mycobacterial AhpC (Fig. 5A). In order to understand the function of this extra loop, a construct with the deletion of the loop residues from 23 to 34 (*Mb*AhpC_Δ23–34_) (Fig. 5A, *red dashed box*) was generated. Oxidized- and reduced (presence of 2 mM DTT) *Mb*AhpC_Δ23–34_ were purified using the purification protocol following wt *Mb*AhpC (Fig. 5B and C). Both the oxidized as well as the reduced form of *Mb*AhpC_Δ23–34_ eluted at 16 ml, resembling the oxidized dimeric form of *Mb*AhpC. Furthermore, no significant change in the hydrodynamic diameter of both the oxidized (6.5 ± 1.9 nm, 25.5% polydispersity) and reduced (5.9 ± 1.8 nm, 28.5% polydispersity) *Mb*AhpC_Δ23–34_ was observed in DLS experiments (Fig. 5D). These results demonstrate that the amino acid stretch 23–34 of *Mb*AhpC is essential for the assembling of the dodecameric ring of reduced *Mb*AhpC. This is underlined by electron micrographs of *Mb*AhpC_Δ23–34_ (Fig. 5E), in which the reduced *Mb*AhpC_Δ23–34_ particles resembled those of oxidized *Mb*AhpC (Fig. 3E). Furthermore, as reflected in Fig. 5F, the *Mb*AhpC_Δ23–34_ mutant did not show significant activity in the NADPH-oxidation assay, indicating that no electron transfer occurred in the catalytic cycle and demonstrating that the N-terminal residues 23–34 are also crucial in maintaining the enzymatic activity.

### Substitutions of N-terminal residues of *Mb*AhpC provide insights into their enzymatic role

**Figure 6:**
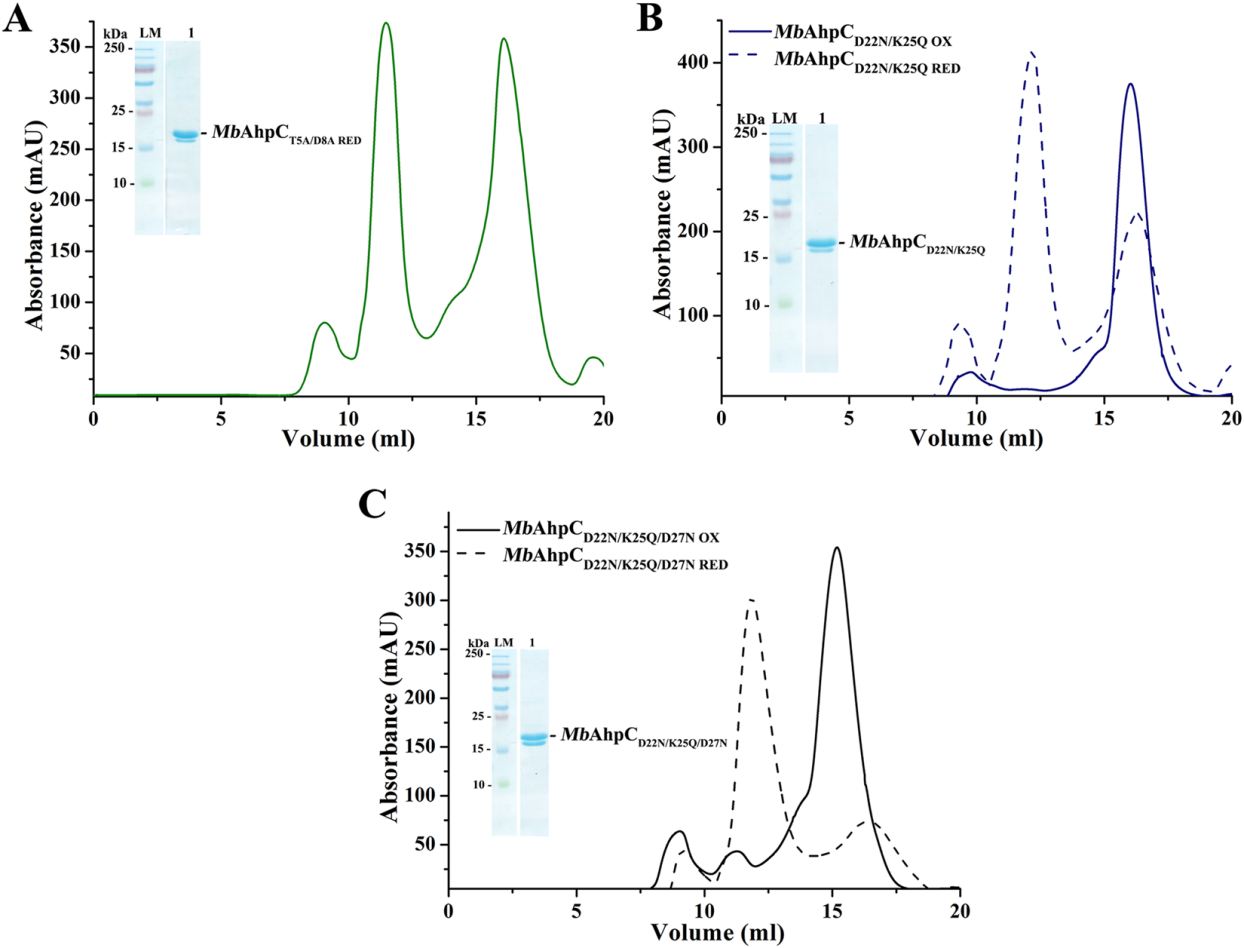
Protein purification of *Mb*AhpC double and triple mutants. (**A**) The recombinant and reduced *Mb*AhpC_T5A/D8A_ eluted in an equal proportion of oligomer (12 ml) and dimer (16 ml) on a Superdex 200 HR 10/30 column and showed high purity on a 17% SDS gel (*inset*). The recombinant oxidized and reduced double mutant, (**B**) *Mb*AhpC_D22N/K25Q_, and triple mutant, (**C**) *Mb*AhpC_D22N/K25Q/D27N_, showed an SEC elution profile resembling wt *Mb*AhpC. Majority of the oxidized recombinant protein (*solid blue and black line*) eluted at approximately 16 ml while the reduced recombinant protein eluted (*dashed blue and black line*) at 12 ml. Both reduced recombinant proteins revealed high purity on a 17% SDS gel (*inset*).

To specify critical residues of the N-terminus in more depth, the polar residues T5, D8, D22, K25 and D27 have been substituted in the double and triple mutants, *Mb*AhpC_T5A/D8A_, -AhpC_D22N/K25Q_, and -AhpC_D22N/K25Q/D27N_, respectively (Fig. 6A–C). All mutants were purified according to the protocol of wt *Mb*AhpC and behaved similar to wt *Mb*AhpC with respect to the elution profile and oligomer formation under oxidized and reduced conditions (Fig. 6B and C), with the exception of the double mutant *Mb*AhpC_T5A/D8A_, which showed a close to 1:1 ratio of dimer and dodecamer formation in reduced condition (Fig. 6A). These differences are also reflected in the NADPH-oxidation assay (Fig. 5F), where mutant *Mb*AhpC_D22N/K25Q_ revealed a similar activity profile like the wt protein, and the *Mb*AhpC_D22N/K25Q/D27N_ showed only a slight increase in activity, while the activity of mutant *Mb*AhpC_T5A/D8A_ dropped significantly. The *K*_*m*_ and *k*_*cat*_ of the mutant *Mb*AhpC_T5A/D8A_ were determined to be 1.65 ± 0.32 µM and 0.009 ± 0.001 s^−1^, respectively, (Fig. 2E, Table 1) resulting in a catalytic efficiency (*k*_*cat*_ */K*_*m*_ (H_2_O_2_)) of 5.44 × 10^3^ M^−1^ s^−1^. Finally, the mutant *Mb*AhpC_T5A_ was generated. The DLS-profile in Supplementary Figure S1 revealed that the single substitution of T5 to an alanine alters partially the change from a dodecamer formation of the reduced form to a mix of a dimer and higher oligomer form of the mutant. In comparison to the effect of the double mutant *Mb*AhpC_T5A/D8A_ (Fig. 6A), this also indicates that both residue T5 and D8 are important for the dodecamer formation of reduced *Mb*AhpC.

### NMR titration experiment of *Mb*TrxC with *Mb*AhpC

**Figure 7:**
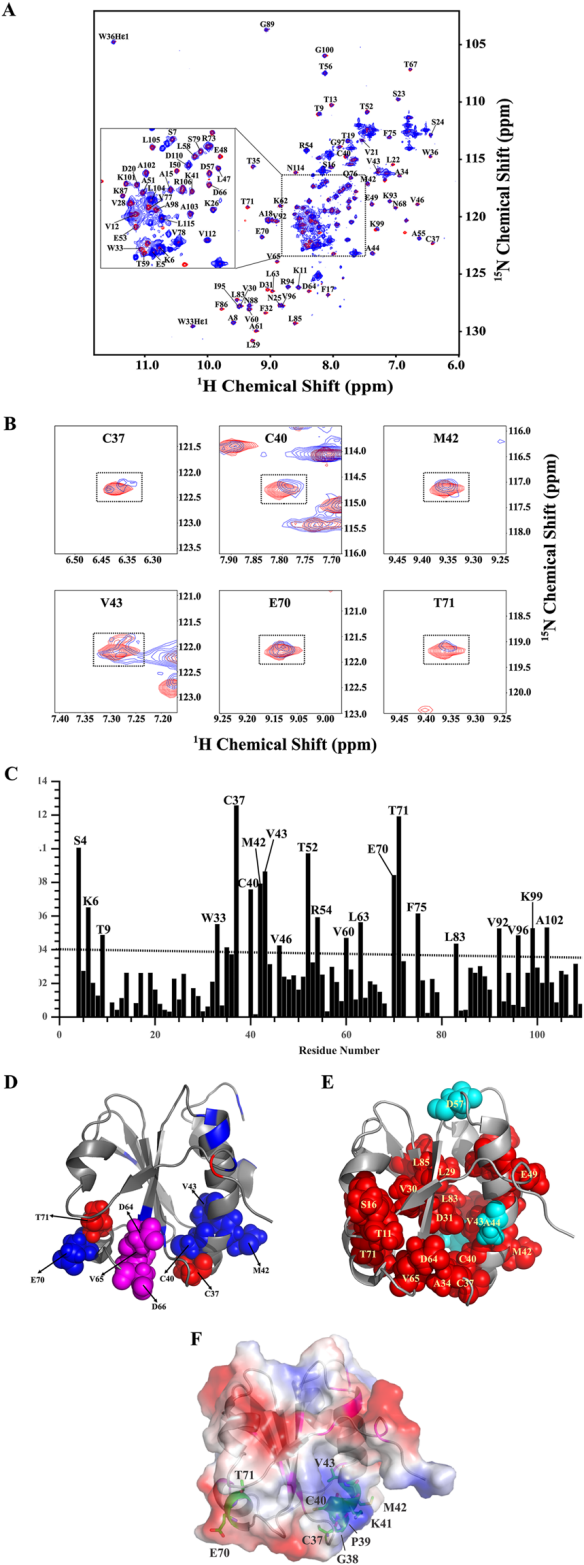
Overlayed ^1^H-^15^N HSQC-spectra of *Mb*TrxC upon titration with *Mb*AhpC at a molar ratio 1:2. (**A**) Superimposition of the ^1^H-^15^N HSQC spectrum of *Mb*TrxC alone (*red*) and in the presence of *Mb*AhpC (*blue*). ^1^H-^15^N HSQC-spectra were obtained on a Bruker avance 700 MHz NMR spectrometer at 298 K. Backbone resonance assignments (based on the BMRB, accession code: 17242)^23^ are indicated in a one-letter amino acid code and sequence number. (**B**) Selected sections for some residues show cross-peaks undergoing significant chemical shift perturbation. (**C**) Molecular interaction between *Mb*TrxC and *Mb*AhpC. A plot of chemical shift perturbations after addition of *Mb*AhpC into *Mb*TrxC at a molar ratio 1:2. Differences of chemical shifts were calculated using the following formula, Δδ = [(^1^H_free_ − ^1^H_bound_)^2^ + (^15^N_free_ − ^15^N_bound_)_2_)]^1/2^. (**D**) Ribbon representation of *Mb*TrxC mapped by CSP results; the residues having CSP more than 0.1 are represented in *red*, while those showing CSP between 0.05 and 0.1 are shown in *blue*. Candidates for interacting residues with *Mb*AhpC are represented as sphere model and are labeled. Additional candidates (D64-D66) based on the linewidth analysis are represented as *magenta* sphere. (**E**) Ribbon and sphere representation of *Mb*TrxC mapped by NMR linewidths analysis; the residues showing increased in linewidth of more than 150% for NH are represented in *red*, while those showing increased in linewidth of more than 120% of ^15^N only are shown in *cyan*. (**F**) Electrostatic potential at the surface of mycobacterial TrxC (PDB ID: 2L4Q)^23^ with the epitopes C37-V43 and E70-T71 shown as stick representation in *green* and the residues involved in indirect interaction as *magenta*.

Since the data above demonstrate clearly that mycobacterial TrxC provides electrons for the reduction of AhpC and thereby the reduction of H_2_O_2_ and *t*-bOOH, a series of NMR titration experiments were performed to examine the molecular interaction between *Mb*TrxC and –AhpC. ^1^H-^15^N-HSQC spectra of ^15^N-labelled *Mb*TrxC were collected at three different molar ratios (1:0, 1:0.5, 1:1 and 1:2) of *Mb*TrxC and *Mb*AhpC (Fig. 7A). During titration experiments, we observed the disappearance or the decrease of some of the cross peak intensities in the ^1^H-^15^N HSQC spectrum (Fig. 7A), slight changes of ^15^N- and HN-resonances for some residues and gradual line broadening with increasing molar ratios (Fig. 7B). This demonstrates the binding of *M*bTrxC with *Mb*AhpC. A plot of chemical shift perturbations after the addition of *Mb*AhpC to labeled *Mb*TrxC at a molar ratio of 1:2 is shown in Fig. 7C. Significant changes (>0.1) in chemical shift were observed for the backbone resonances of regions including C37-V43 and E70-T71. These residues are considered as directly involved in the interaction with *Mb*AhpC. Other residues including S4, K6, T9, W33, T52, R54, V60, L63, F75, L83, V92, V96, K99, A102 and L115 are affected indirectly by the protein-protein interaction. In addition, the NMR spectra showed also gradual line broadening and increased linewidths after the addition of *Mb*AhpC (Fig. 7A), which supports the slow molecular tumbling and fast T2 relaxation times caused by the formation of the *Mb*TrxC-AhpC complex. The average increased linewidths before and after binding of *Mb*AhpC to *Mb*TrxC is ~120% of NH and ~90% of ^15^N resonances, respectively. The significant changes (>150% of NH and/or 120% of ^15^N) in linewidths were observed for the backbone resonances of T13, S16-A18, L29, V30, D31 (disappeared), A34, W36, C37, C40, M42-A44, V46, E49-I50, D57, A61, D64-D66, T71-A72, F75, V77, S79, L83, L85 (disappeared), G97-K99, A103, L105 and R106 (Fig. 7E). Interestingly, residues in TrxC identified as the direct binding site (C34-V43 and T71) as well as additional residues including D64-D66 also showed significant changes in linewidths, suggesting that these residues are crucial in the formation of the *Mb*TrxC-AhpC ensemble. As shown in Fig. 7D, the identified residues of *Mb*TrxC involved in AhpC binding form an epitope based on the CSP and NMR linewidth analysis data, including the catalytic cysteine residues, which provide an ideal close proximity for electron transfer during the reduction of the catalytic cysteine-cysteine-center of mycobacterial AhpC. Moreover, the electrostatic charge distribution of *Mb*TrxC (Fig. 7F) showed that the region of amino acids C37-V43 is mostly positively charged and the region E70-T71 is negatively charged, thereby allowing several charged interactions between mycobacterial TrxC and AhpC. Furthermore, NMR titration experiments using *Mb*AhpC_T5A/D8A_ or -AhpC_D22N/K25Q/D27N_ also showed disappearance of resonances or slight changes in chemical shifts for residues located around the binding epitopes (Supplementary Figure S2), which demonstrate that the mutations on these residues do not affect the interaction between *M*bTrxC and *Mb*AhpC.

### Model of the mycobacterial TrxC-AhpC complex

**Figure 8:**
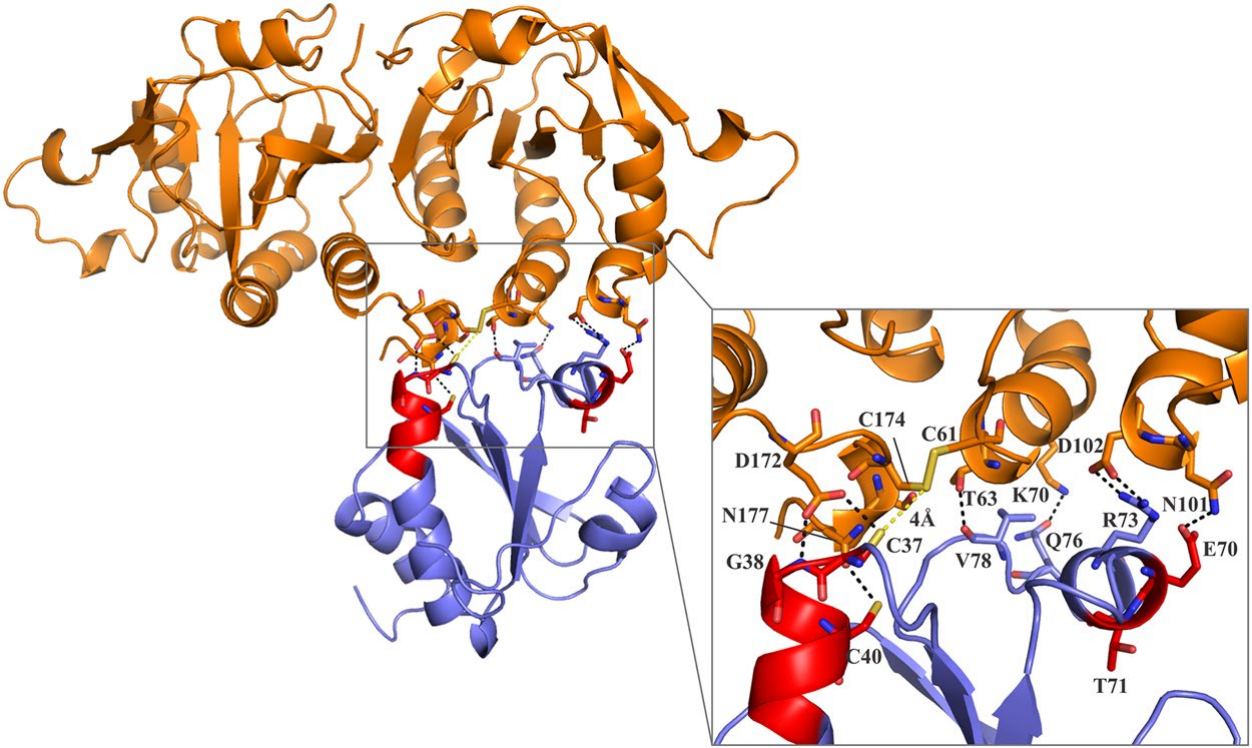
A model of mycobacterial TrxC-AhpC complex based on the NMR titration experiments. TrxC (PDB ID: 2L4Q)^23^ is shown in blue and *Mt*AhpC_C176S_ (PDB ID: 2BMX)^13^ in *orange* color. (*Inset*) A closer view of the various interactions between mycobacterial TrxC and AhpC in the model complex.

In order to model the TrxC-AhpC complex, the obtained NMR restraints for TrxC were used to drive a HADDOCK-docking simulation as described under Materials and Methods. For AhpC, the binding region is chosen to be the redox-center that contains the peroxidatic cysteine C61 of one subunit and the resolving cysteine C174 of the other subunit in the dimer interface^10^. The top scoring HADDOCK model from the highest scoring cluster was selected (HADDOCK score: −35 ± −5.9; restraint violation energy: 27.7 ± 14.55 Kcal/mol; buried surface area: 1124.8 ± 41.4 Å; Z-score: −0.1). The resulting TrxC-AhpC complex model (Fig. 8) indicated the spatial proximity of the two catalytic sites, with C37 of TrxC at a distance of 4 Å from C174 of AhpC. In this complex model, TrxC has several distributed interactions with one of the subunits of the dimer *Mt*AhpC_C176S_ and with the other subunit concentrating near the very C-terminal residues of the catalytic site. Several hydrogen bonding interactions occur in this concentrated region, such as between D172 of AhpC and the TrxC residues C37 and G38, and N177 of AhpC with C40 of TrxC. The interactions of residues C37, G38 and C40 of the C37-V43 region are nicely confirmed by the NMR titration experiment above. Furthermore, in the other interfacial region, a strong salt-bridge interaction occurs between R73 and D102 of TrxC and AhpC, respectively, which is closer to the E70-T71 epitope, wherein E70 interacts with N101 of AhpC through a hydrogen bond. In addition, K70 and T63 of AhpC also have hydrogen bonding interaction with Q76 and V78 of TrxC. Inclusion of additional residues that showed increased linewidths in the NMR experiments above for the *Mb*TrxC-*Mb*AhpC interactions in the docking, did not produce a better complex model.

## Discussion

The mycobacterial peroxidase system appears to play an important role in *M*. *tuberculosis* resistance against the oxidative and nitrosative stress exerted by the host immune response^18^. These enzymes thus represent suitable targets for novel anti-tuberculosis strategies, in particular for INH-resistant *M*. *tuberculosis* strains, where AhpC is thought to compensate for the decreased catalase-peroxidase KatG activity^18, 19^. We demonstrate that *Mb*AhpC, which is identical in amino acid composition to *Mt*AhpC, catalyses NADPH-driven hyperoxide reduction together with the mycobacterial TrxC, resulting in efficient H_2_O_2_ reduction. The *K*_*m*_ values determined for *Mb*Trx-AhpC with H_2_O_2_ (2.57 ± 0.36 μM) or *t*-bOOH (2.23 ± 0.49 µM) as a substrate are similar (Table 1), with an increased catalytic efficiency of *Mb*AhpC with *t*-bOOH (Table 1). In comparison, the *K*_*m*_ of *Mb*Trx-AhpC with *t*-bOOH (2.23 ± 0.49 µM) is slightly lower compared to the reported *Mt*Trx-AhpC complex with *t*-bOOH as a substrate (5.6 μM)^15^. The difference may in part be caused by the fact that NADH has been used as an electron donor in the studies of *Mt*Trx-AhpC^15^ instead of the natural substrate NADPH (Fig. 1A) in the studies presented with *Mb*AhpC. Together with the results of Jaeger *et al*.^15^, the data presented confirm that mycobacterial TrxC reduces efficiently AhpC and refute the proposal that only AhpD and not the thioredoxin reductase would be a redox partner of mycobacterial AhpC^1^. Because of its efficiency, the mycobacterial Trx-AhpC system becomes essential to protect the pathogenic bacterium against oxidative stress and therefore, the interaction of both TrxC-AhpC are important to shed light into their electron transfer and the interacting residues enabling the TrxC-AhpC complex formation.

Comparison of the *Mt*AhpC_C176S_ mutant structure with other peroxiredoxins showed that the helix α1 is flexible and is involved in a rigid-body movement to position the peroxidatic cysteine (C61) either in contact with the resolving cysteine (C174), or in the active center (Fig. 8)^13^. A small rearrangement of three phenylalanine side chains (F51, F68, F108) has been proposed to cause such displacement, and an internal cavity would be created due to the movement of the helix α1, which was proposed as a potential drug binding site^13^. The presented NMR-titration as well as the molecular docking studies extend the molecular model of electron transfer inside mycobacterial AhpC and the electron donor TrxC. In the derived *Mt*TrxC-AhpC complex model, TrxC makes two crucial interactions with helix α1 of AhpC, whereby residues Q76 and V78 of TrxC have hydrogen bonding interaction with K70 and T63 of AhpC, respectively (Fig. 4). As F68 is present on helix α1, we speculate that during complex formation, the interactions mentioned above might induce the rearrangement of the phenylalanine side chains, which will aid in the helical displacement. Moreover, in the TrxC-AhpC complex model, C37 of TrxC is closer to C174 (4 Å) than C61 (5.5 Å) of AhpC. This favors an alternative mechanism, which was proposed for the interaction of mycobacterial AhpC with AhpD by Guimarães *et al*.^13^ for three cysteine preoxiredoxins. It was proposed that after the condensation reaction of peroxidatic cysteine (C_P_61) in helix α1 with the resolving cysteine (C_R_174), the disulfide bond between C61-C174 is reduced by C176 and a second intramolecular disulphide bond between C174 and C176 occurs and subsequently, the external thiol (coming from AhpD or TrxC) attacks either one of the resolving cysteines.

**Figure 9:**
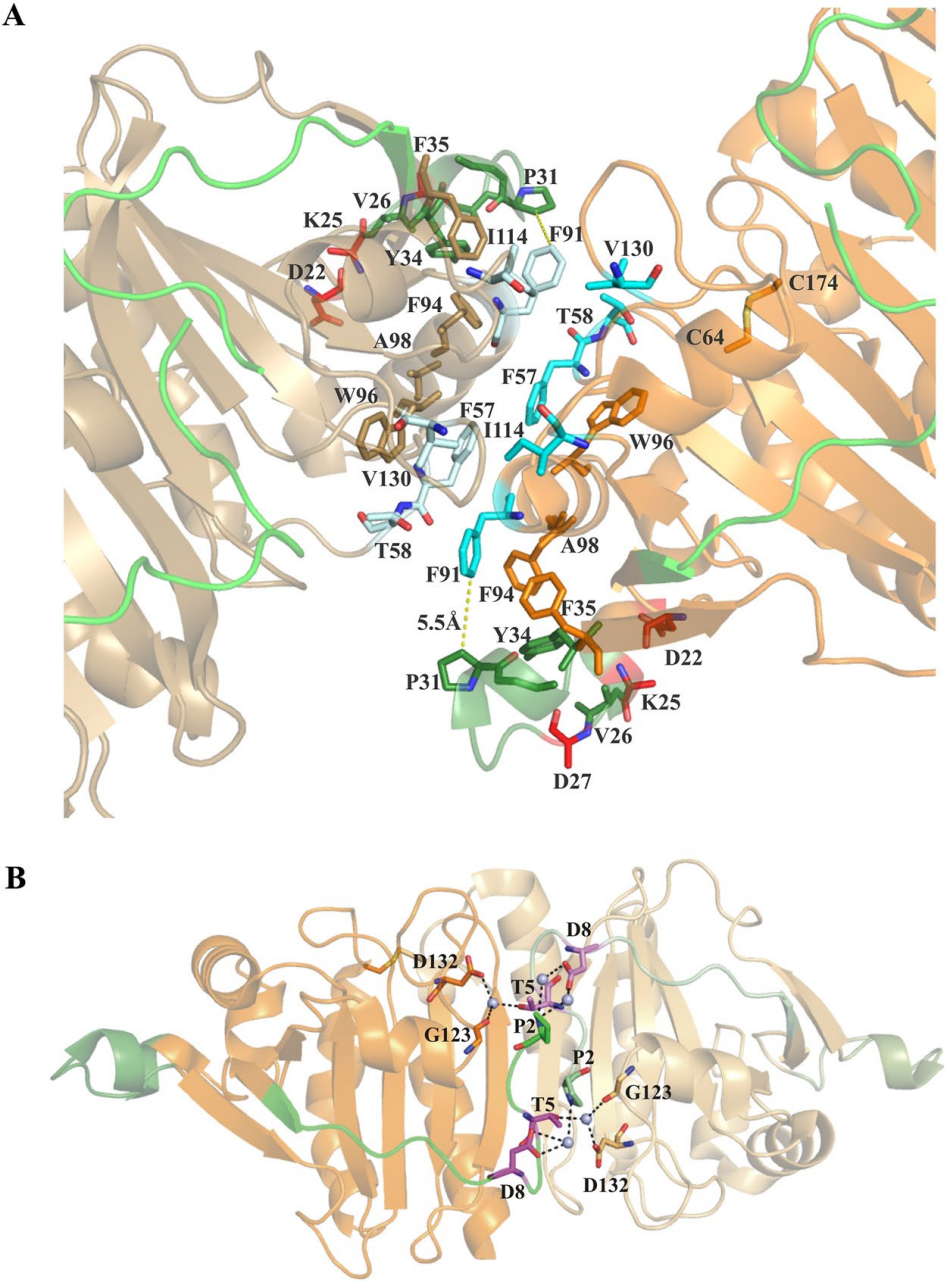
(**A**) Dimer-dimer interface of the *Mt*AhpC_C176S_ structure (PDB ID: 2BMX)^13^. The first monomer is shown in *orange* and the second monomer is shown in *sand* color. The N-terminal 1–15 residues are shown in *green* and the extra loop (23–34) in the N-terminal is shown in *forest green*. The hydrophobic residues F57, T58, F91, I114 and V130 that stabilize the interface are shown in *cyan*. The mutants D22, K25 and D27 are highlighted in *red*. (**B**) Critical residues in the dimer interface of mycobacterial AhpC (PDB ID: 2BMX)^13^. The mutant residues T5 and D8 are shown in *magenta*. The subunits are shown in *orange* with the second in lighter shade. The N-terminal 1–15 residues are shown in *green* and the extra loop (23–34) in the N-terminal is shown in *forest green*. The water molecules involved in the water mediated interaction are shown as *light blue* spheres.

We also visualized the dodecameric ring formation of the reduced *Mb*AhpC (Fig. 3D). So far, the crystallographic structure of the oxidized *Mt*AhpC_C176S_ mutant revealed a dimer in the asymmetric unit, and the dodecameric ring formation of the oxidized *Mt*AhpC_C176S_ mutant structure was proposed to be forced by crystal packing forces^13^. The electron microscopy data of *Mb*AhpC in solution demonstrate that the mycobacterial protein undergoes a transfer from a dimer to a dodecameric oligomer under oxidized and reduced conditions, respectively. Deletion of and substitution inside the unique N-terminus of mycobacterial AhpC highlight the importance of this region in maintaining protein stability, redox-modulated oligomer formation and enzyme activity. Interestingly, the N-terminal deletion mutants *Mb*AhpC_16–195_ and *Mb*AhpC_41–195_ are unstable and formed undefined oligomers, indicating the importance of the unique N-terminal residues for the stability of mycobacterial AhpC redox- oligomarization. Moreover, the deletion mutant *Mb*AhpC_Δ23–34_ demonstrated that the mutant exists as a dimer in the oxidized and the reduced state and has no activity, highlighting that the N-terminal residues 23–34 are important for maintaining not only the oligomerization but also the enzymatic activity. In the *Mt*AhpC_C176S_ mutant structure, the dimer-dimer interface of the dodecamer is mainly stabilized by the hydrophobic interactions of the residues F57, T58, F91, I114 and V130 of the interacting monomers^13^. The hydrophobic environment of F91 that faces the solvent region is occluded by the extra loop of the N-terminal amino acids 23–34, which is at a distance of 5.5 Å (Fig. 9A). The hydrophobic residues of the extra loop V26, P31, Y34 and F35 are positioned in such a way to create an ample hydrophobic environment for helix α2, that holds F91, F94, A98 and W96 (a highly-conserved residue directly associated with the redox-induced dimer-decamer switch in peroxiredoxins)^13^. We speculate, that by removing this extra loop, the hydrophobic environment might become destabilized for F91 and for helix α2, which in-turn might destabilize the dimer-dimer interface. Therefore, the oligomer formation would be hindered and finally the enzymatic activity would be affected. Interestingly, the double and triple mutants *Mb*AhpC_D22N/K25Q_ and *Mb*AhpC_D22N/K25Q/D27N_ did not affect the enzymatic activity, since the substitutions are made for the polar residues of the extra loop that faces the solvent region and which does not significantly contribute to the hydrophobic environment of the dimer-dimer interface (Fig. 9A).

Furthermore, the double mutant *Mb*AhpC_T5A/D8A_ at the very N-terminus reduced enzymatic activity by 2.73 × 10^3^ M^−1^s^−1^ (33%) when compared to wt *Mb*AhpC, indicating the critical role of these residues. The unique N-terminal stretch (amino acids 1–15) spans the complete subunit AhpC, and the very N-terminal residues bridge the functional dimer interface in the *Mt*AhpC_C176S_ structure^13^. The residue T5 of one subunit makes water-mediated hydrogen bonding interactions with D132 and G123 of another subunit (Fig. 9B), confirming its importance in dodecamer formation as shown in the *Mb*AhpC_T5A_ mutant (Supplementary Figure S1). In addition, amino acid D8 of one subunit has water-bridge interaction with P2 of another subunit in the functional dimer interface. Due to the substitution of these residues to a non-polar alanine in the *Mb*AhpC_T5A/D8A_ mutant, these interactions might be abolished, and we speculate, that this might weaken the dimer interface, thus resulting in the decreased activity.

In summary, we show that *Mb*TrxC-AhpC forms an NADPH-dependent peroxidase ensemble for efficient reduction of H_2_O_2_ inside the mycobacterial antioxidant defense system. Identification of the amino acids involved in TrxC and AhpC interaction provide not only a new epitope for structure-guided drug design but also shed light into the electron transfer from TrxC to the catalytic cysteines of AhpC and possible rearrangements of side chains of residues F51, F68, and F108 of helix α1. AhpC undergoes a redox-modulated dimer to dodecamer formation, in which the unique mycobacterial N-terminal stretch of AhpC place a fundamental role as revealed by a variety of N-terminal mutants of *Mb*AhpC. Since the dodecamer ring formation is essential for the proper enzyme catalysis, the important roles of this unique N-terminal stretch in dimer and oligomer formation as well as enzyme activity makes this N-terminus a novel epitope for drug design.

## Materials and Methods

### Cloning, production and purification of *Mb*AhpC, and its mutants as well as *Mb*TrxB2 and -TrxC

The coding region for the residues 1–195 of *Mb*AhpC was amplified from the genomic DNA of *M*. *bovis* (BCG strain), which is identical in its amino acids sequence to *Mt*AhpC (Fig. 1C), by polymerase chain reaction (PCR) using the forward primer 5′-ATG ACC ATG GTC ATG CCA CTG CTA AC-3′ and the reverse primer 5′-AAC CAG AGG ATC CTT AGG CCG AAG-3′. The coding region for the mutant *Mb*AhpC_16–195_ was amplified using the forward primer 5′-CCT CCA TGG TCA CCG CTC TCA TCG GCG GTG AC-3′ and the reverse primer 5′-AGC CGG ATC CTT AGG CCG AAG CCT TGA GGA CTT C-3′. The coding region for the mutant *Mb*AhpC_41–195_ was amplified using the forward primer - 5′ CCT CCA TGG TCA CCG CTC TCA TC 3′ and the reverse primer - 5′ AGC CGG ATC CTT AGG CCG AAG CCT T3′. The amplified PCR-products were gel-extracted and ligated into the pET9-d1-His_6_ vector^20^. The mutant *Mb*AhpC_Δ23–34_, -AhpC_T5A_, -AhpC_T5A/D8A_, -AhpC_D22N/K25Q_ and -AhpC_D22N/K25Q/D27N_ were created by In-Fusion-based mutagenesis^21^. The whole pET9-d1-His_6_^20^ plasmid carrying *Mb*AhpC gene was used as the template. The deletion of residues 23 to 34 (*Mb*AhpC_Δ23–34_) was introduced by amplifying the whole plasmid using the primers 5′-GGT GAT AGT GGT GAA GTC ACC GCC GAT GAG AGC GGT GAG CTG-3′ and 5′-TTC ACC ACT ATC ACC AGT GAC GAA CAC CCA GGC AAG TGG CGG-3′. For *Mb*AhpC_T5A_, -AhpC_T5A/D8A_, -AhpC_D22N/K25Q_ and -AhpC_D22N/K25Q/D27N_, point mutations were made and the whole plasmid was amplified using the primer sets 5′-TGC TAG CTA TTG GCG ATC AAT TCC CCG-3′ and 5′-CCA ATA GCT AGC AGT GGC ATG ACC ATG-3′, 5′-TGC TAG CTA TTG GCG CTC AAT TCC CCG CCT ACC AG-3′ and 5′-ATT GAG CGC CAA TAG CTA GCA GTG GCA TGA CCA TG-3′, 5′-GGT AAT CTG TCC CAA GTC GAC GCC AAG CAG-3′ and 5′-GAC TTG GGA CAG ATT ACC GCC GAT GAG AGC G-3′, and 5′-GTC AAT GCC AAG CAG CCC GGC-3′ and 5′-GGC ATT GAC TTG GGA CAG ATT ACC GCC-3′, respectively.

Genomic DNA of *M*. *bovis* was used to amplify *M*. *bovis* thioredoxin (*Mb*TrxC) and thioredoxin reductase (*Mb*TrxB2). *Mb*TrxC was constructed using the forward primer 5′-GCA CAA CGC CAT GGC AAT GAC CGA TTC CGA GAA GT-3′ and the reverse primer 5′-CCG GGA TCC CTA GTT GAG GTT GGG AAC CAC GTC T-3′, and *Mb*TrxB2 was constructed using forward primer 5′-TTA ACC ATG GAT ATG ACC GCC CCG CCT GTC-3′ and reverse primer 5′-TTA TGG ATC CTC ATC GTT GTG CTC CTA TCA ATG CGT CGG-3′. In *Mb*AhpC, -AhpC_16–195_, -AhpC_40–195_, -TrxC and -TrxB2 constructs, the restriction sites *NcoI* and *BamHI* (underlined) were incorporated into the forward and reverse primers, respectively. The amplified PCR-products were purified and ligated into the pET9-d1-His_6_ vector^20^.

The coding sequences of all constructs were verified by DNA sequencing. The final plasmids were subsequently transformed into *E*. *coli* BL21 (DE3) cells (Stratagene). To express the recombinant proteins, the cells were grown in a shaker incubator in LB medium, containing kanamycin (30 μg/ml), for about 4 h at 37 °C until an optical density OD_600_ of 0.6–0.7 was reached. For *Mb*TrxC, cells were cultured in LB medium containing kanamycin (30 µg/ml) and chloroamphenicol (34 µg/ml). To induce the production of proteins, cultures were supplemented with isopropyl β-D-1-thiogalactopyranoside (IPTG) to a final concentration of 1 mM, and incubated for 16–18 hours at 18 °C.

Cells producing recombinant *Mb*AhpC, -AhpC_Δ23–34_, -AhpC_16–195_, -AhpC_41–195_, -AhpC_T5A_, -AhpC_T5A/D8A_, -AhpC_D22N/K25Q_, -AhpC_D22N/K25Q/D27N_, -TrxC and -TrxB2 were lysed on ice by sonication with an ultrasonic homogenizer (Bandelin, KE76 tip) for 3 × 1 min in buffer A containing 50 mM Tris/HCl, pH 7.5, 200 mM NaCl, 0.8 mM DTT and 2 mM Pefabloc^SC^. For oxidized conditions, buffer A without 0.8 mM DTT was utilized instead. After sonication, the cell lysate was centrifuged at 12 000 × *g* for 25 min at 4 °C (Eppendorf, Germany). The resulting supernatant was passed through a filter (0.45 µm; Millipore) and incubated with Ni^2+^-NTA resin pre-equilibrated in buffer A. His-tagged proteins were allowed to bind to the matrix for 1 h at 4 °C by mixing on a sample rotator (Neolab). Subsequently, the protein was eluted with an imidazole gradient (20–600 mM). The fractions containing recombinant proteins were identified by SDS–PAGE^22^ and further purified by size exclusion chromatography (SEC) using a Superdex 200 HR 10/30 column or Superdex 75 HR 10/30 column (GE Healthcare, Sweden), with buffer containing 50 mM Tris/HCl, pH 7.5 and 200 mM NaCl.

#### Enzymatic characterization of mycobacterial AhpC

Peroxide-dependent activity of the various forms of purified recombinant *Mb*AhpC proteins were measured by coupling its activity with NADPH-oxidation (ɛ_280_ = 6220 M^−1^ s^−1^) catalyzed by *Mb*TrxC and *Mb*TrxB2. The peroxidase activity was carried out at 25 °C by monitoring the decrease in NADPH-absorbance at 340 nm for 120 sec using a stopped-flow spectrophotometer SX20 (Applied Photophysics, UK) equipped with a sample handling unit that comprises two drive syringes. The reaction mixture containing 0.1 mM NADPH, 50 mM HEPES buffer pH 7.0, 100 mM ammonium sulfate, 1 mM EDTA, 0.5 µM of *Mb*TrxB2, 4 µM of *Mb*TrxC, and 6 μM of *Mb*AhpC, -AhpC_Δ23–34_, -AhpC_T5A/D8A_, -AhpC_D22N/K25Q_ or -AhpC_D22N/K25Q/D27N_ were loaded into drive syringe 1, and mixed with 50 µM of hydrogen peroxide, which was loaded into drive syringe 2, to initiate NADPH-oxidation. NADPH consumption measured in the absence of the respective proteins was taken as a control, and this background rate was subtracted from the experimental rate to determine the activity due to AhpC. All data reported here is the average of three independent measurements. The characterization of *Mb*AhpC and *Mb*AhpC_T5A/D8A_ peroxidase activity were performed following the above mentioned, except with various increasing concentration of hydrogen peroxide (250 nm–50 μM) or *t*-bOOH (250 nm–30 μM), to initiate NADPH-oxidation. All the experimental curves were created using the program Origin Pro 9.0 (OriginLab Corporation).

#### Dynamic light scattering

Dynamic light scattering (DLS) of the oxidized and reduced (presence of 2 mM DTT) *Mb*AhpC, -AhpC_Δ23–34_ and -AhpC_T5A_ were carried out using a Malvern Zetasizer Nano ZS spectrophotometer. DLS experiments were performed in a low-volume quartz batch cuvette (ZEN2112, Malvern Instruments) using 12 μl of the respective protein solution. After 60 s equilibration time, the backscattering at 173° was collected for all proteins. Scattering intensities were analyzed using the in-built software, Zetasizer to calculate the hydrodynamic diameter (D_H_), size, and volume distribution.

#### NMR titration experiments of *Mb*TrxC with *Mb*AhpC

Samples for NMR experiments were prepared in 20 mM sodium phosphate, pH 6.5, 100 mM NaCl, 0.001% NaN_3_ and 10% D_2_O. All NMR measurements were performed on a Bruker Avance 700 MHz spectrometer, equipped with a 5 mm z-axis-gradient cryogenic probe at 298 K. The resonance assignments of ^15^N and NH resonances were achieved based on the previously reported NMR chemical shift data of mycobacterial TrxC from the BioMagnetic Resonance Bank (BMRB, accession code: 17242)^23^. To study the interaction between *Mb*TrxC and *Mb*AhpC, unlabeled *Mb*AhpC or -AhpC_T5A/D8A_ or -AhpC_D22N/K25Q/D27N_ was titrated stepwise into a solution with 0.1 mM reduced ^15^N labeled *Mb*TrxC. ^1^H-^15^N HSQC spectra were recorded with 2048 complex data points in in *t*_*2*_ and 240 *t*_*1*_ increments at molar ratios of 1:0, 1:0.5, 1:1 and 1:2 (*Mb*TrxC:*Mb*AhpC), respectively. Spectral width of 2270 and 11160 Hz were employed in F1 (^15^N) and F2 (^1^H), respectively. All data were processed with NMRPipe^24^ and NMRDraw^24^, and spectrums were analyzed using SPARKY^25^. In order to determine the interacting residues of *Mb*TrxC with *Mb*AhpC, the previously reported structure of reduced form of mycobacterial TrxC (PDB code: 2L4Q)^23^ was used, and the respective residues were visualized by PyMOL^26^.

#### Molecular docking of mycobacterial AhpC with TrxC

The interaction identified from the NMR titration experiments were used to perform docking studies to model the complex of mycobacterial AhpC and TrxC using the HADDOCK webserver^27^. The coordinates of mycobacterial AhpC (PDB ID: 2BMX)^13^ and TrxC (PDB ID: 2L4Q)^23^ were obtained from the Protein Data Bank. Residues experiencing significant perturbation during titration were used as restrains. In the case of TrxC, the directly interacting residues are specified as “active” and the neighbouring residues as “passive”, whereby for AhpC, the “active” region is the redox-center and the “passive” region is the surrounding region. The submitted subunits are placed in space with an approximate distance of 25 Å and a complex is formed through rigid body energy minimization, which resulted initial in 1000 structures. The top 200 lowest energy models from the above minimization are subjected to a simulated annealing in torsion-angle space procedure and subsequent flexible refinement in explicit solvents. Models which fulfilled the interfaced ligand RMSD cutoff of 5 Å were clustered into ten groups. The best model in the highest scoring cluster was then selected for analysis. The model was visualized using the PyMOL software package^26^.

#### Electron microscopy and 2D image analysis of *Mb*AhpC and its mutants

*Mb*AhpC and *Mb*AhpC_Δ23–34_ were diluted to a final concentration of 80 μg/ml in buffer containing 50 mM Tris/HCl, pH 7.5 and 200 mM NaCl. In the case of the reduced protein, DTT was added to a final concentration of 2 mM. A volume of 4 μl of protein sample was applied to a 30 second glow discharged carbon coated copper TEM grid and negatively stained with 2% (v/v) uranyl acetate. Electron micrographs were recorded on a FEI Tecnai T12 transmission electron microscope (FEI) equipped with a 4 K CCD camera (FEI) operated at a voltage of 120 kV at a calibrated magnification of 66,350x. 37 micrographs were recorded at 0° angle for the reduced *Mb*AhpC. A total of 4054 particles for reduced *Mb*AhpC were selected for 2D projection analysis. The selection criteria of particles were, (i) the clear visibility of single molecules and (ii) separation from neighboring particles. Particles were picked using EMAN2^28^ and processed with RELION 1.4^29^.

## Electronic supplementary material

Supplementary figures
