# Supplementary material for: AhpC of the mycobacterial antioxidant defense system and its interaction with its reducing partner Thioredoxin-C
Source: Sci Rep. 2017 Jul 11;7:5159. doi: 10.1038/s41598-017-05354-5 (PMC5505994; doi:10.1038/s41598-017-05354-5)
Supplement: Supplementary file 1 — Supplementary figures [file 41598_2017_5354_MOESM1_ESM.pdf]

AhpC of the mycobacterial antioxidant defense system and its interaction with its reducing partner Thioredoxin-C

Chui Fann Wong[1], Joon Shin[1], Malathy Sony Subramanian Manimekalai[1], Wuan Geok Saw[1], Zhan Yin[1,2], Shashi Bhushan[1,2], Arvind Kumar[1], Priya Ragunathan[1], and Gerhard Grüber[1,*]

[1] Nanyang Technological University, School of Biological Sciences, 60 Nanyang Drive, Singapore 637551, Republic of Singapore

[2] NTU Institute of Structural Biology, Nanyang Technological University, Republic of Singapore

[*] To whom correspondence should be addressed: Prof. Dr. Gerhard Grüber, Tel.: + 65 - 6316 2989; Fax: + 65 - 6791 3856, E-mail: ggrueber@ntu.edu.sg

Keywords: *Mycobacteria*, Tuberculosis, Redox homeostasis, Reactive oxygen species, Oxidative stress, Alkylhydroperoxide reductase

Supplementary Figure 1. DLS analysis of reduced *Mb*AhpC (*red*) and the reduced mutant *Mb*AhpC$_{T5A}$ (*green*) revealed a smaller oligomer in *Mb*AhpC$_{T5A}$ as compared to wt *Mb*AhpC. This further illustrates the importance in T5 in maintaining the dodecamer formation.

Supplementary Figure 2. Selected sections of the overlaid ^{1}H-^{15}N-HSQC spectra of free *Mb*TrxC (*red*), as well as *Mb*TrxC-*Mb*AhpC$_{T5A/D8A}$ (*blue*) and *Mb*TrxC-*Mb*AhpC$_{D22N/K25Q/D27N}$ (*green*) complexes at a molar ratio of 1 (*Mb*TrxC) : 2 (*Mb*AhpC mutants). The residues undergoing disappearance of resonance or chemical shift perturbation are marked as box and labelled.

(A)

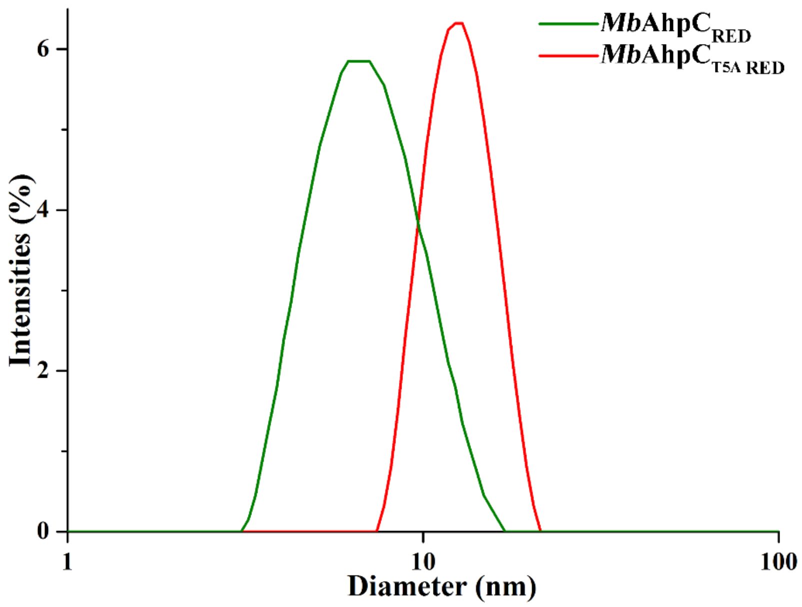

Supplementary Figure 1

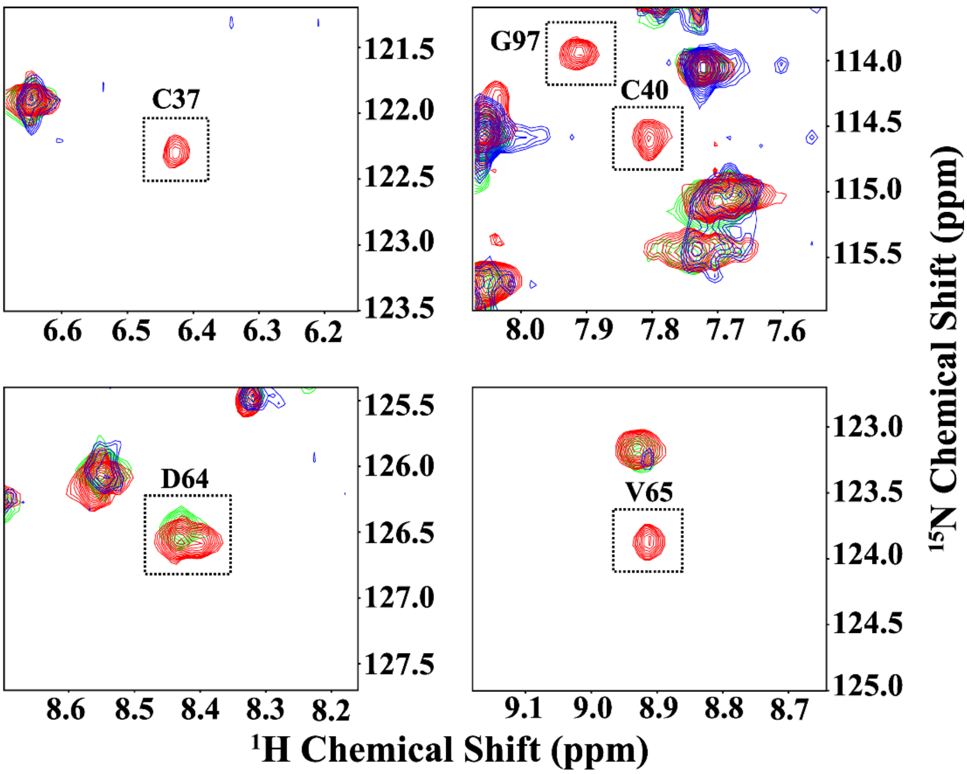

Supplementary Figure 2
